# Infections associated with immunotherapeutic and molecular targeted agents in hematology and oncology. A position paper by the European Conference on Infections in Leukemia (ECIL)

**DOI:** 10.1038/s41375-019-0388-x

**Published:** 2019-01-30

**Authors:** Georg Maschmeyer, Julien De Greef, Sibylle C. Mellinghoff, Annamaria Nosari, Anne Thiebaut-Bertrand, Anne Bergeron, Tomas Franquet, Nicole M. A. Blijlevens, Johan A. Maertens, on behalf of the European Conference on Infections in Leukemia (ECIL)

**Affiliations:** 1 Department of Hematology, Oncology and Palliative Care, Klinikum Ernst von Bergmann, Charlottenstrasse 72, 14467 Potsdam, Germany; 2 Department of Internal Medicine and Infectious Diseases, Saint-Luc University Hospital, Université Catholique de Louvain, Brussels, Belgium; 3 Assistance Publique-Hôpitaux de Paris (AP-HP), Department of Hematology, Henri Mondor Teaching Hospital, Créteil, France; 4 Department I of Internal Medicine, University Hospital of Cologne, Cologne, Germany; 5 Cologne Excellence Cluster on Cellular Stress Responses in Aging-Associated Diseases (CECAD), University of Cologne, Cologne, Germany; 6 Department of Hematology, ASST Grande Ospedale Metropolitano Niguarda, Milan, Italy; 7 Department of Hematology, Université Grenoble Alpes, Grenoble, France; 8 Department of Pneumology, Université Paris Diderot, APHP Saint-Louis Hospital, Paris, France; 9 Department of Radiology, Hospital de Sant Pau, Barcelona, Spain; 10 Department of Hematology, Radboud University Medical Centre, Nijmegen, Netherlands; 11 Department of Hematology, University Hospitals Leuven, Leuven, Belgium; 12 Department of Microbiology and Immunology, University of Leuven, Leuven, Belgium

**Keywords:** Epidemiology, Immunopathogenesis

## Abstract

A multitude of new agents for the treatment of hematologic malignancies has been introduced over the past decade. Hematologists, infectious disease specialists, stem cell transplant experts, pulmonologists and radiologists have met within the framework of the European Conference on Infections in Leukemia (ECIL) to provide a critical state-of-the-art on infectious complications associated with immunotherapeutic and molecular targeted agents used in clinical routine. For brentuximab vedotin, blinatumomab, CTLA4- and PD-1/PD-L1-inhibitors as well as for ibrutinib, idelalisib, HDAC inhibitors, mTOR inhibitors, ruxolitinib, and venetoclax, a detailed review of data available until August 2018 has been conducted, and specific recommendations for prophylaxis, diagnostic and differential diagnostic procedures as well as for clinical management have been developed.

## Introduction

Immunotherapeutic agents and small molecules for molecular targeted treatment have profoundly changed the landscape of antineoplastic therapy in hematology and oncology. Their impact on innate and adaptive immunity is not yet completely understood. Given to heavily pretreated patients and combined with other anticancer treatment modalities, they may be associated with unexpected, potentially serious infections. However, in heavily pretreated patients, particularly those with chronic lymphocytic leukemia and other indolent B-cell lymphomas, it may be difficult to identify a causal relationship between those infections and drugs administered for lymphoma treatment. As immune-related autoinflammatory reactions are typical adverse events (irAE) occurring in many of these patients, differential diagnostic efforts to distinguish those reactions from infections are crucial. In patients affected by irAE, immunosuppressive treatment will often be required, resulting in secondary infections. This complex scenario calls for a high alertness to formerly unexpected clinical complications among hematologists and oncologists using these newer antineoplastic agents. At the same time, unjustified attribution of infections to these agents and recommendations for routine antimicrobial prophylaxis should be avoided. This position paper updates our current knowledge of infections associated with these agents and provides recommendations for a rational clinical management of prevention and treatment of infections in patients treated with immunotherapeutic and molecular targeted antineoplastic agents.

Immunotherapeutic and molecular targeted antineoplastic agents including checkpoint inhibitors, idelalisib, mTOR inhibitors, and, to a lesser extent, ibrutinib have been associated with drug-related pneumonitis. This pneumonitis is clinically indistinguishable from infectious pneumonias, and the diagnosis relies on the exclusion of differential diagnoses. The specific management of drug-related pneumonitis includes drug withdrawal and consideration for corticosteroids according to the severity.

## Methods

A group of experts in hematology and oncology, infectious diseases (including virology), pulmonology, diagnostic radiology and hematopoietic stem cell transplantation from six European countries was nominated by the ECIL organization committee in 2016. Immunotherapeutic and molecular targeted agents, which have become available for clinical use within the past decade, including brentuximab vedotin, blinatumomab and immune checkpoint inhibitors as well as HDAC inhibitors, ibrutinib, idelalisib, mTOR inhibitors, ruxolitinib and venetoclax, were addressed. Other agents approved during the last 10 years (such as obinutuzumab, newer proteasome inhibitors, pomalidomide, daratumumab, elotuzumab or inotuzumab ozogamicin) were excluded, because thorough literature searches did not identify relevant infection risks attributable to these agents, or no new signals as compared to older drugs from the same class of agents were found, or because their approval was extremely limited (such as mogamulizumab). A systematic literature review including research papers, case reports, published study results and meta-analyses or review articles was conducted using drug- and class-based search strings: “(agent)” OR “(class)” AND “mode of action” OR “approval” OR “study” OR “infection” OR “infectious” OR “toxicity” OR “adverse events” OR “viral” OR “bacterial” OR “Pneumocystis” OR “fungal” OR “pneumonia” OR “pneumonitis” OR “CNS” OR “hepatitis” OR “cytomegalovirus” OR “immune-related” OR “prophylaxis”. The group compiled an extensive slide set including mode of action, state of approval, impact on innate and adaptive immunity, reported infectious complications and recommendations for clinical practice. After mail-based and face-to-face group discussions, the revised and consented slides were presented to the plenary of the seventh ECIL conference in Sophia Antipolis, France, on 22 and 23 September 2017. The plenary decided to abandon grading and strength of recommendations, because the data comprised represent a “moving target” with a rapidly growing body of reports. In order to provide a critical and detailed summary of the current knowledge in the field, the result should be published as an ECIL position paper.

All co-authors have been actively involved in the preparation and discussion of this manuscript.

The agents included are addressed in the following sequence: (1) immunotherapeutic drugs and (2) molecular targeted drugs/drug classes, both in alphabetical order.

A summary of available data as well as ECIL recommendations is listed in Table 1.

**Table 1:** Summary of drug characteristics, reported infectious complications and ECIL recommendations for clinical practice

Class of agents	Agent	Impact on immune system	Infectious events	ECIL recommendations
CD19-directed CD3 bispecific T-cell engager	Blinatumomab	B-cell aplasia; hypogammaglobulinemia; neutropenia	No clear evidence of increased infection rate	•Consideration of IgG substitution in case of serious infection
Anti-CD30 antibody	Brentuximab vedotin	Poorly defined; impairment of memory cell generation and survival; transient neutropenia	Pneumocystis pneumonia; CMV and HBV reactivation; JC virus-associated PML	•CMV monitoring; •No routine systemic antimicrobial prophylaxis; •High alertness to PML
Immune checkpoint inhibitors	Ipilimumab (anti-CTLA4); Nivolumab, pembrolizumab, atezolizumab and others (anti-PD-1/anti-PD-L1)	No direct immunosuppression	Frequent immune-related auto-inflammatory complications; infections due to anti-inflammatory/immunosuppressive agents	•High alertness to infections if anti-inflammatory/immunosuppressive agents are required; •Pneumocystis prophylaxis if glucocorticosteroid medication exceeds 3−4 weeks
Bruton Tyrosine Kinase Inhibitor	Ibrutinib	Toll-like receptor-mediated recognition of infectious agents; Maturation, recruitment and function of innate immune cells, including neutrophils, monocytes and macrophages; Regulation of NLRP_3_ inflammasome activation	Slight increase in bacterial, fungal and viral infections, particularly in heavily pretreated patients; Cerebral aspergillosis in patients treated for lymphoma with brain involvement	•Update protective vaccinations before ibrutinib treatment; •Increased alertness to infections; •At signs of infection, diagnostics including bacterial, viral and fungal pathogens; •No routine systemic antimicrobial prophylaxis
Phosphatidylinositol- 3-kinase inhibitor	Idelalisib	Decrease in number and function of regulatory T cells; Inhibition of NK cell and neutrophil inflammatory response; Neutropenia	Slight increase in Pneumocystis pneumonia	•Anti-Pneumocystis prophylaxis (see label); •Check CMV serostatus and consider CMV monitoring; •At signs of infection, consider immune-related adverse reaction
Histone deacetylase inhibitors	Vorinostat, panobinostat, romidepsin	Inhibition of toll-like receptor-mediated dendritic cell and macrophage function (sensing, phagocytosis, cytokine production, adhesion)	No clear evidence of increased infection rate	•HBV screening, consideration of antiviral prophylaxis in HBsAg- or anti-HBc-positive patients
mTOR inhibitors	Sirolimus, temsirolimus, everolimus	Inhibition of T-cell proliferation, antigen-presenting cells, B cells, neutrophil granulocytes	No clear evidence of increased infection rate	•High alertness of infections; •No routine antimicrobial prophylaxis; •At signs of pulmonary infection, consider immune-related adverse reaction
Janus kinase inhibitor	Ruxolitinib	Inhibition of dendritic cell and CD4^+^ T-cell function; Reduction of regulatory T cells; NK cell inhibition	Marginally increased risk of opportunistic infections; Occasional HBV reactivation	•Careful monitoring for infections; •HBV screening, prophylactic entecavir in HBsAg- or anti-HBc-positive patients; •MTB screening in patients-at-risk
BCL2 inhibitor	Venetoclax	Neutropenia	15−20% grade ≥ 3 infections	•Management according to neutropenia; •Consider G-CSF; •At combination with posaconazole, venetoclax dose reduction by ≥75%

*NLRP3, NLR* (nucleotide-binding domain, leucine-rich repeat) family, pyrin domain containing 3, *G-CSF* granulocyte-colony stimulating factor, *HBV* hepatitis B virus, *CMV* cytomegalovirus, *JC* John Cunningham polyomavirus, *PML* progressive multifocal leucoencephalopathy, *IgG* immunoglobulin G, *MTB* *Mycobacterium tuberculosis*

## Immunotherapeutic agents (blinatumomab, brentuximab vedotin, immune checkpoint inhibitors): characterization, impact on immunity, reported infectious complications and recommendations for clinical practice

### Blinatumomab

Blinatumomab is a bispecific T-cell engaging (BiTE) antibody, approved for the treatment of Philadelphia-chromosome negative relapsed or refractory B-precursor acute lymphoblastic leukemia (R/R ALL) [1]. It is made of a single-chain anti-CD19 antibody attached by a flexible linker peptide to a single-chain anti-CD3 antibody. It induces a close contact between effector T-cells and CD19-positive cells, with subsequent T-cell activation and targeted lysis of CD19-positive cells [2]. CD19 is expressed in all B-cell lineage leukemia, and a majority of B-cell lineage lymphomas. In normal cells, it is expressed all along B-cell differentiation, with the exception of pluripotent stem cells and plasma cells [3].

Even at very low doses, blinatumomab has been shown to induce a rapid and sustained decrease of measurable peripheral B cells [4, 5]. All B cells are depleted, including CD19-negative plasma cells as a consequence of precursors and CD19-positive plasmablast clearance. A decrease of immunoglobulin (IgG, IgA, IgM) levels has been reported with slow recovery at long-term follow-up [6]. The exact impact on preexisting immunity is unknown, and, although anticipated, a correlation with increased risk of infection has not been proven. Interestingly, CTL019, another anti-CD19 antibody-based immunotherapy, also induces B-cell aplasia and hypogammaglobulinemia, but stable titers of several vaccine- and pathogen-specific serum immunoglobulin G and A were noted [7].

The second known immunosuppressive effect of blinatumomab is neutropenia, with grade 3 neutropenia reported in 18−32% of patients [1]. This rate is variable according to the context, and in advanced ALL, grade 3 or higher neutropenia has been shown to be 20% less frequent with blinatumomab than with chemotherapy (37.8% vs. 57.8%) [8]. In a follow-up of blood counts in patients treated with blinatumomab for R/R ALL, median neutrophil count decreased in responders from 1.8 × 10^3^/μL to 0.6 × 10^3^/μL on day 7, but did not decrease anymore during subsequent cycles. Febrile neutropenia was not reported in clinical studies in the context of MRD-positive ALL or NHL, but grade 3 events occurred in up to 24% of patients in R/R ALL [5, 9].

Overall, infections of all grades were reported in 45% of treated patients, with grade 3 or higher in 27% [1]. This infection rate must be interpreted cautiously in the context of advanced hematologic malignancies and heavily pretreated patients. Results of the phase-3 TOWER trial were confirmatory, showing lower grade 3 or higher infection rates in the blinatumomab group compared to chemotherapy (34.1% vs. 52.3%) [8]. Invasive fungal infections have been reported rarely, and a recent review has identified ten reported cases associated with blinatumomab use in ALL, again probably linked to the context [10]. Venous catheter-related infections constitute an important concern, blinatumomab being administered by continuous infusion for 2−4 weeks [11].

In conclusion, blinatumomab has not been associated with a high infectious risk, but some practical recommendations can be made. A particular attention should be put on central venous lines management, and clinicians should be alert for the risk of catheter-related infections. Immunoglobulin level monitoring and supplementation in case of low IgG concentration is recommended, particularly in patients with a history of serious infections [12].

### Brentuximab vedotin

Brentuximab vedotin (BV) is an antibody-drug conjugate (ADC) of a chimeric anti-CD30 antibody and the synthetic anti-tubulin monomethyl auristatin E (MMAE). The ADC binds to the membrane glycoprotein CD30, inducing subsequent intracytosolic release of MMAE after internalization and proteolytic cleavage of the dipeptidic ADC linker [13]. The drug is approved for the treatment of relapsed or refractory CD30-positive classical Hodgkin’s lymphoma (HL), as consolidation therapy after autologous hematopoietic stem cell transplantation in HL, and for relapsed or refractory anaplastic large T-cell lymphoma (ALCL) [14].

CD30 is a member of the Tumor Necrosis Factor receptor superfamily, which is highly expressed by Reed-Sternberg cells in HL and by malignant cells in ALCL. It shows a variable level of expression on the surface of malignant cells in other NHL. CD30 has a low level of expression in normal cells, mainly restricted to a small subset of activated B-, T- (CD4- and CD8-positive) and natural killer (NK) cells [15]. CD30 has been shown to play a complex role in immune response, which has not been fully elucidated yet. Among other, it is thought to help to maintain CD8-positive effector cell activity during antigenic challenge [16]. It is involved in the transition from effector cells to central memory cells and the survival of memory cells. This role in the control of memory cells could help to control pathogens such as listeria and mycobacteria [17]. Other mechanisms have also been suggested implying CD30-positive cells in antimycobacterial immune response, and those cells have been found in positive tuberculin skin tests and TB-infected tissues [18]. By killing CD30-positive cells, BV may induce an immune dysbalance facilitating those infections, but it should be noted that no clinical association has been demonstrated as yet.

Transient dose-dependent neutropenia is a commonly observed side effect of BV. When given as a single agent in relapsed or refractory HL or ALCL or for consolidation therapy after autologous HSCT, BV was shown to induce grade ≥3 neutropenia in 20−29% of patients. However, febrile neutropenia was extremely rare [19–21]. In contrast, myelosuppression appears to be an important concern when BV is used in combination with chemotherapy. In a phase 3 study evaluating BV + AVD vs. ABVD in stage III or IV HL, the use of BV was associated with a higher risk of grade ≥3 neutropenia (58% vs. 45% respectively) and with higher rates of febrile neutropenia, mainly during the first cycle (9% vs. 4% respectively). The risk was reduced by primary G-CSF prophylaxis [22].

Overall, BV does not appear to be associated with a high risk of infectious complications. Phase 3 studies did not show a higher infection rate in the BV group compared to controls [21, 23]. A slightly higher overall infectious risk was described in the BV + AVD group than in the ABVD group, but was mitigated by G-CSF administration [22].

However, some specific concerns have been raised about particular pathogens or situations.

Pneumonia has been reported in up to 10% of BV-treated patients [14], with even higher rates when combined with chemotherapy [24]. *Pneumocystis* pneumonia (PcP) was rare (0.1−1%) [14]. Noninfectious pulmonary toxicity has been reported [25], but is more likely to be attributable to the coadministration of bleomycin, so that this combination has become contra-indicated [9]. Large phase 3 studies did not show pulmonary toxicity when BV was not combined with bleomycin [21, 23].

Varicella Zoster Virus (VZV) and Herpes Simplex Virus (HSV) infections are described as common side effect of BV, with an incidence of 1−10% [149]. Extensive or disseminated diseases have been reported [26, 27]; however, a clear causal relationship is doubtful because of the impact of many other risk factors in affected patients.

Although not described in pivotal studies, two case series of cytomegalovirus (CMV) reactivation under BV have been published, questioning the true incidence of this event and a possible causal relationship. In allogeneic stem cell recipients, 5 CMV viremias among 25 patients treated with BV for HL recurring after allogeneic HSCT were reported. Three patients required treatment and one died in the setting of CMV reactivation [28]. Another report described three cases of CMV reactivation with retinitis among 32 lymphoma patients treated with BV. Patients responded to therapy, but two out of three relapsed after BV rechallenge [29].

Concerns about a risk of JC virus (John Cunningham polyoma virus) infection in patients treated with BV have been raised early after the approval of BV. A boxed warning was inserted in the drug label in 2012. At that time, two proven and one probable case of progressive multifocal leukoencephalopathy (PML) had been reported among 2000 patients treated worldwide [30]. Additional cases have been described since then [31], with a total of 15 cases reported until July 2015 to the FDA’s Adverse Event Reporting System. The case fatality rate was 33.3% [32]. It must be kept in mind that those reported cases do not prove a causal relationship, as lymphoid malignancy, multiagent chemotherapy or hematopoietic cell transplantation are PML risk factors [33]. While there is no estimated PML incidence known for patients with HL, the rate for those with NHL is estimated to be 8.3 (95% CI 1.71–24.24) per 100,000 person-years [34].

For clinical practice, no specific recommendation can be made with regards to antimicrobial prophylaxis. G-CSF prophylaxis should be considered when BV is used in combination with chemotherapeutic agents. PcP prophylaxis is not required, if BV is given without concomitant treatment [35]. The same rule applies to HSV and VZV prophylaxis [36]. CMV should be taken into consideration in case of symptoms compatible with infection, but no prophylaxis, routine monitoring or preemptive therapy can be recommended for patients undergoing treatment with BV. For JC virus, no prophylaxis is available, but clinicians should be alert and prompt a complete work-up in case of new-onset neurological symptoms suggestive of PML. BV should be withheld until PML has been excluded. In case of confirmation, BV should be discontinued with the aim to restore immunity against JC virus. In some cases this may be complicated by an immune reconstitution inflammatory syndrome [37]. However, in the case of BV-associated PML, due to underlying disease and previous or concurrent treatments, immune recovery is uncertain and the clinical course is unpredictable. PML cases should be notified to local competent authorities, in order to document this rare possible association.

### Immune checkpoint inhibitors

Immune checkpoint inhibition (ICI) has introduced a new era of cancer therapy [38]. It represents a novel therapeutic concept, as the primary target is the crosstalk between immune cells and cancer cells in the tumor microenvironment. Two immune checkpoints are currently targeted by approved drugs: the programmed death 1 (PD-1)/PD-ligand 1 (PD-L1) axis as well as cytotoxic T-lymphocyte antigen-4 (CTLA-4). Blockade of the PD-1 or PD-L1 pathway has been shown to exert therapeutic activity in patients with Hodgkin lymphoma [39], head and neck squamous cell carcinoma [40], advanced melanoma [41, 42], non-small cell lung carcinoma [43, 44], and renal cell carcinoma [45, 46]. Further indications may follow soon [47, 48]. The anti-CTLA-4 antibody ipilimumab was the first immune-checkpoint antibody approved for the treatment of patients with advanced melanoma due to its survival benefit compared to standard chemotherapy [41, 49, 50].

PD-1 is a cell surface coinhibitory receptor expressed on T- and B-lymphocytes, monocytes and NK-cells after activation [51]. To date, PD-L1 (B7-H1) and PD-L2 (B7-DC) have been identified as ligands of PD-1. Both ligands are expressed on antigen-presenting cells, and PD-L1 is additionally detected on the surface of various nonhematopoietic cells including tumor cells. The binding of PD-1 to its ligands results in an inhibition of T-cell receptor signaling on activated T-lymphocytes. In addition, the PD-1/PD-L1 pathway is a key component in the development and maintenance of self-tolerance [52]. Furthermore, there is increasing evidence that the PD-1/PD-L1 pathway is important in the pathogenesis of different tumors by inhibiting, and thus limiting antitumor immune response [53–58].

CTLA-4 is a second immune-checkpoint expressed during priming of T cells [41].

Inhibition of the PD-1/PD-L1 as well as the CTLA-4 pathway non-specifically activates the immune system resulting in imbalance. A broad spectrum of immune-related adverse events (irAE) may occur, involving the gut, skin, endocrine glands, liver, lungs [59], and possibly other organs [60, 61]. One rare irAE is neutropenia caused by autoantibodies against neutrophils. Two cases have been reported, one of them associated with a *Staphylococcus aureus* infection [62, 63]. IrAE often require immunosuppressive medication, which in turn increases the susceptibility to severe infections [47, 64, 65], resulting in up to 7.3% opportunistic infections in patients affected [66]. Few data (mostly preclinical) report on higher risk for tuberculosis [67], histoplasmosis [68], and listeriosis [69, 70] due to ICI. At present, the incidence of infection is undetermined and the role of prophylactic antiviral and antifungal therapy in this setting is undefined.

Prophylaxis of PcP should be considered if secondary immunosuppression is given for at least 3 weeks [35, 71, 72] As patients receiving ICI are at increased risk of infection due to their underlying malignancy, it is recommended that they receive all appropriate vaccines at the earliest possible moment [73]. Future identification of biomarkers predicting AE, e.g. microbiota in the gut, may help to facilitate preemptive treatment [74, 75].

## Molecular targeted agents (ibrutinib, idelalisib, HDAC inhibitors, mTOR inhibitors, ruxolitinib, venetoclax): characterization, impact on immunity, reported infectious complications and recommendations for clinical practice

### Ibrutinib

The inhibition of Bruton’s tyrosine kinase (BTK) is a crucial strategy for treating B-cell malignancies. Ibrutinib, an irreversible inhibitor of BTK, is now approved for the treatment of chronic lymphocytic leukemia (B-CLL) [76, 77], mantle cell lymphoma [78], marginal zone lymphoma [79], small lymphocytic lymphoma [80] and Waldenström’s macroglobulinemia [81]. Ibrutinib is also the first approved therapy for the treatment of chronic graft-versus-host disease after failure of one or more lines of systemic therapy [82]. BTK has been widely characterized as a critical mediator of B-cell receptor signaling that regulates B-cell survival, activation, differentiation, and interaction with the environment [83]. Germline mutations in the gene encoding for BTK result in an almost complete absence of mature B cells and hypogammaglobulinemia, the hallmark of X-linked (Bruton’s) agammaglobulinemia [84]. Hence, BTK is essential in the development and functioning of adaptive immunity. However, BTK also plays a major role in innate immunity: (a) in Toll-like receptor-mediated recognition of infectious agents, (b) in maturation, recruitment and function of innate immune cells, including neutrophils, monocytes and macrophages, and (c) in regulating NLRP_3_ inflammasome activation [85]. Thus, targeting BTK with ibrutinib in a population already characterized by an immune dysregulation (e.g. CLL) will most likely result in an increased risk of infection.

Collectively, the randomized pivotal trials demonstrate that upper respiratory tract infections are the most common infectious complications in ibrutinib-treated patients, albeit mostly self-resolving [76–81]. Pneumonia is the most common serious infectious event. The frequency and pattern of infections appears to reflect what is typically seen in this B-cell malignancy population, rather than a drug-specific adverse event profile. Infectious complications are considerably fewer and less severe in treatment-naïve (TN) compared with relapsed/refractory (R/R) patients, i.e., 13% vs. 51% ≥ grade 3 infections: pneumonia (6% vs. 25%), sepsis (0% vs. 7%), cellulitis (0% vs. 5%) sinusitis (0% vs. 5%) and bacteremia (0% vs. 4%) [86]. The infectious morbidity appears to decrease over time; grade ≥ 3 infections are observed more frequently during the first 6 months of therapy, often during the first 2−3 months. This trend is related to the extended time from last chemotherapy in R/R cases, early response and disease control, and the immunomodulating potential of ibrutinib. In addition, prolonged ibrutinib treatment results in a partial reconstitution of the humoral immunity with stabilization or improvement of immunoglobulin levels and of the normal B-cell populations [87].

Not unexpectedly in these often heavily pretreated patients, opportunistic infections have been sporadically reported, including cases of cryptococcal disease (meningoencephalitis/disseminated disease) [88–91], (miliary) tuberculosis [92, 93], endemic mycosis, PML (in rituximab pretreated patients) caused by JC virus [94–96], Epstein−Barr Virus (EBV)-driven hemophagocytic syndrome [97], and reactivations of Hepatitis B virus (HBV) [98, 99].

Following a report of five cases of PcP in a cohort of 96 patients [100], concern has risen that ibrutinib therapy could increase the risk of PcP, although no other study has reported a frequency above 1%. These PcP cases occurred in previously untreated CLL patients receiving ibrutinib monotherapy and presented in a “nontypical” way [100]: (a) patients were asymptomatic or had only mild, often chronic respiratory symptoms; (b) there was no long-term use of steroids or other immunosuppressive drugs; (c) chest computed tomography scan revealed nontypical multifocal nodular infiltrates; (d) CD4^+^ T-cell counts were high (>500 per microliter), (e) and no patient required intravenous therapy, adjunctive steroid treatment or mechanical ventilation. Of note, only one of these five cases was confirmed by immunofluorescence, still considered the gold diagnostic standard for PcP. The FDA Division of Pharmacovigilance recently reviewed 13 additional cases of confirmed and presumed PcP submitted to the FDA Adverse Event Reporting System [101]. Contrary to the previous case series, ten of these cases had refractory underlying disease with prior exposure to other immunosuppressive agents and six cases reported concomitant use of such agents. Thus, although the inhibitory effect of ibrutinib on interleukin-2-inducible kinase makes an increased risk for PcP biologically plausible, PcP prophylaxis is not routinely recommended; its risk-benefit should be outweighed in the context of diminished T-cell immunity due to previous (e.g. fludarabine-cyclophosphamide-rituximab therapy) or concomitant therapy [35].

Among thousands of patients with a variety of B-cell malignancies treated with ibrutinib, invasive mold infections have been reported only sporadically. The frequency of invasive yeast and mold infections in the clinical studies was low, ranging from 0 to 3.2% [1–6]. More recently, a retrospective French survey reported 27 cases of invasive aspergillosis from 16 centers [102]. Most cases occurred early-on within a median of 3 months after starting ibrutinib for relapsed/refractory disease. Cerebral involvement was frequent (40%). Unfortunately, the survey did not report a denominator, and the majority of patients had at least one additional factor, aside from hypogammaglobulinemia, that increased their risk for fungal infections [102]. During a 5-year period (2012−2016), invasive fungal infection (including pulmonary and disseminated aspergillosis, pulmonary cryptococcosis, and PcP) developed in 4.2% of ibrutinib-treated patients at the Memorial Sloan Kettering Cancer Center [103]. Experimental use of single-agent ibrutinib in patients with primary central nervous system lymphoma was associated with a 5−27% frequency of invasive aspergillosis, including cerebral disease [104, 105]. Clearly BTK plays a role in innate fungal immune surveillance (as demonstrated in *Btk*^−/−^ mice studies [104]) via a series of mechanisms mentioned before [85]. Obviously ibrutinib impairs that fungal immune surveillance, thereby contributing to the complex “net state of immunosuppression”, although the increased susceptibility to fungal disease in ibrutinib-treated patients remains primarily dictated by the status of the underlying lymphoid malignancy, the combined action with other immunosuppressive therapies and the environmental exposure to fungal pathogens [103, 106]. However, these reports underscore the need for heightened awareness and vigilance to identify any change in fungal epidemiology in view of the rapidly growing availability of novel therapeutic agents with immunosuppressive characteristics. Pending further epidemiological data, routine mold-active prophylaxis is currently not recommended (outside the setting of severe graft-versus-host disease post-allogeneic stem cell transplantation, where ibrutinib may become a treatment option). It must be kept in mind that mold-active azoles interfere with ibrutinib elimination by inhibiting the CYP3A4 enzyme system, potentially increasing the risk of adverse events [107]. However, as the indications for ibrutinib use continue to expand, better identification of risk factors for invasive fungal disease may define populations in which monitoring and antifungal prophylaxis can be studied as potential preventive strategies [103].

Guidelines recommend vaccination against influenza and pneumococcal disease in patients with B-cell malignancies [73, 108]. However, recent prospective data demonstrate that ibrutinib may dramatically impair adequate serological responses to vaccination [109, 110]; hence, clinicians may consider vaccinating patients before the initiation of antineoplastic therapy.

Finally, there have been sporadic reports of pneumonitis in patients receiving ibrutinib [111]. These cases present early (1−4 months) after initiation of therapy and are clinically indistinguishable from infectious complications (e.g. PcP or viral pneumonitis). Diagnosis is established by ruling out other differential diagnoses; treatment includes ibrutinib withdrawal and corticosteroids.

### Idelalisib

Idelalisib is a selective inhibitor of adenosine-5’-triphosphate in the phosphatidylinositol-3-kinase delta (PI3Kδ). It is approved since 2014 in combination with rituximab for the treatment of relapsed chronic lymphocytic leukemia (B-CLL) and for first-line therapy of B-CLL with del17p or TP53 mutation and as a monotherapy for refractory follicular lymphoma.

Phosphatidylinositol 3-kinase (PI3K) comprises a group of related lipid enzymes regulating pleiotropic downstream effector functions. Class I PI3Ks are heterodimers of regulatory and catalytic subunits with four different isoforms, α, β, γ and δ, involved in cell proliferation, survival, and motility [112, 113]. The α and β isoforms are widely expressed in many tissues, whereas γ and δ isoforms are restricted to hematopoietic cells. In B lymphocytes, the δ isoform (PI3Kδ) plays a central role in normal B-cell development and function, transducing signals from B-cell receptor as well as from receptors for various cytokines, chemokines and integrins [114, 115]. PI3Kδ signaling pathways are frequently hyperactive in many B-cell malignancies [116–118], so that the inhibition of δ isoform-specific PI3K signaling is a promising approach for the therapy of B-cell lymphoma. Idelalisib blocks PI3Kδ-AKT (protein kinase B) signaling and promotes apoptosis of B-lymphocytes.

Few reports describe a higher risk of opportunistic infections in patients treated with idelalisib, particularly PcP and CMV infections, even in the setting of normal neutrophil counts and absence of profound lymphocytopenia. It was hypothesized that PI3K inhibitors cause an increased susceptibility to infections through impairment of granulocyte activation [119]. Four trials have been published on monotherapy [120–123], three in combination with anti CD20 [124–126] and four with other combinations [127–130]; three of them were stopped early because of excess adverse event rates (hepatotoxicity and pneumonitis) [128–130].

Regarding bacterial infections, no increased risk was found to be associated with idelalisib. For clinical practice, no specific recommendations for antibacterial prophylaxis can be given. Sehn et al. published a retrospective analysis of 2198 patients receiving idelalisib alone or in combination with co-therapy (anti-CD20 antibody or bendamustine + rituximab) and patients receiving only co-therapy (anti-CD20 ± bendamustine) [131]. The overall incidence of PcP was 2.5% in patients on idelalisib ± co-therapy vs. 0.2% in patients receiving anti-CD20 antibody alone or in combination with bendamustine (relative risk, 12.5). A correlation between CD4 counts (e.g., <200 cells/µL) and an increased risk of PcP was not observed. Only 1.2% of patients receiving anti-*Pneumocystis* prophylaxis developed this complication, as compared to 3.5% of those without prophylaxis, and among the 20% of patients in whom PcP prophylaxis was administered, no deaths occurred. Thus, there is a small, but increased risk of PcP during treatment with idelalisib. Prophylaxis with trimethoprim-sulfamethoxazole is included in the label now, and the European Society of Clinical Microbiology and Infectious Diseases study group for infections in compromised hosts (ESGICH) suggests PcP prophylaxis during idelalisib therapy and for 2−6 months after its discontinuation [132]. From our perspective, PcP prophylaxis is recommended, but based on weak evidence [133, 134].

Cytomegalovirus reactivations are notified in randomized trials for 52 of 2204 patients (2.4%) treated with idelalisib (https://www.ema.europa.eu/documents/variation-report/zydelig-h-c-003843-a20-1439-0023-epar-assessment-report-article-20_en.pdf) [123, 126, 127, 135]. The incidence rate is higher when idelalisib is combined with bendamustine (13/207 patients; 6.3%) (https://www.ema.europa.eu/documents/variation-report/zydelig-h-c-003843-a20-1439-0023-epar-assessment-report-article-20_en.pdf) [127, 135]. CMV serostatus must be defined for all patients before treatment initiation. For CMV-negative patients, CMV-negative or filtered blood products are recommended and CMV antigen or PCR should be checked at least every 4 weeks. In case of positive PCR/antigen with increasing viral load or symptoms consistent with CMV disease, ganciclovir or valganciclovir treatment is recommended and idelalisib should be discontinued [134].

### Histone deacetylase (HDAC) inhibitors (panobinostat, vorinostat, romidepsin)

HDAC inhibitors are used for epigenetic treatment affecting the coiling and uncoiling of DNA around histones, involving histone acetyl transferases and histone deactetylases [136]. For use in clinical hematology, panobinostat (in combination with bortezomib and dexamethasone for recurrent multiple myeloma), vorinostat (T-cell lymphoma progressive, persistent or recurrent on or following two systemic therapies) and romidepsin (treatment of relapsed cutaneous T-cell lymphoma and peripheral T-cell lymphoma) are approved.

HDAC inhibitors exert a plethora of inhibitory effects on innate immunity, in particular on toll-like receptor-mediated dendritic cell (DC) and macrophage function such as sensing, phagocytosis, cytokine production or adhesion [137], resulting in increased microbial susceptibility and reduced inflammatory response [138]. However, in controlled clinical trials on HDAC inhibitor use in patients with multiple myeloma, malignant lymphoma (T cell, B cell or Hodgkin’s), acute myeloid or lymphoblastic leukemia or myelodysplastic syndrome, no significant increase in infection rates or fever have been observed in comparison with control groups [139–150]. A notable rate of asymptomatic interstitial pneumonitis has been reported from a clinical trial on panobinostat used for treatment of Waldenström’s Macroglobulinemia [151]. From observations outside clinical hematology, a potential use of HDAC inhibitors for improved clearance of Human Immunodeficiency Virus has been postulated [152–155].

For clinical practice, no clear evidence of HDAC inhibitor-attributable increase in the risk of infection or infection-related mortality has been reported. Hence, there is no rationale for specific prophylaxis and for specific diagnostic procedures in case of fever in hematologic patients under treatment with HDAC inhibitors. Considering their negative impact on inflammatory response, screening for HBV and consideration of prophylactic drug treatment in case of reactivation risk may be recommended. In patients with active infection, HDAC inhibitor treatment should be withheld. In case of cough and/or dyspnea, drug-related interstitial lung disease should be taken into consideration. HDAC inhibitor use in HIV-positive patients with hematologic malignancies does not seem to increase the risk of HIV activation.

### mTOR inhibitors (sirolimus, temsirolimus, everolimus)

Inhibitors of the mammalian target of rapamycin (mTOR) are approved for immunosuppression post solid organ transplantation and the treatment of mantle cell lymphoma, breast cancer, neuroendocrine tumors and renal cell cancer. Sirolimus, temsirolimus and everolimus are available for clinical application.

mTOR is acting as a serine/threonine protein kinase in the PI3k/AKT signaling pathway of growth factor receptors such as epidermal growth factor (including HER-2), vascular endothelial growth factor and insulin-like growth factor-1 receptor. Immunosuppression and impaired wound healing may result from inhibition of T-cell proliferation, antigen-presenting cells, B cells, neutrophil granulocytes, mast cells and stromal cells [156, 157]. A meta-analysis of published reports on 5436 patients treated with mTOR inhibitors showed a nonsignificantly increased risk of all-grade leukopenia and neutropenia [158], while another meta-analysis of 3180 mTOR inhibitor-treated patients [159] demonstrated a relative risk of all-grade and high-grade infections of 2.00 (95% CI, 1.76−2.28, *p* < 0.001) and 2.60 (95% CI, 1.54−4.41, *p* < 0.001), respectively, as compared with patients in the control arms of the studies. Infections mainly affect the respiratory tract (61.7%), genitourinary tract (29.4%) and skin/soft tissue (4.2%). A difference in incidences or risks between everolimus and temsirolimus or between different tumor types (renal cell carcinoma vs. others) was not observed. Among respiratory tract infections, no increase in the risk of specific types of pneumonia such as PcP, invasive mold or CMV infection was found to be associated with mTOR inhibition [160]. Urinary tract infections caused by polyomavirus or CMV were even less frequently observed in 4930 renal transplant recipients receiving mTOR inhibitors as compared with those treated with mycophenolate for preventing graft rejection [161]. A meta-analysis of 14 clinical trials on post-transplant mTOR inhibitor treatment confirmed a lower rate of CMV reactivation among heart transplant recipients [162].

Studies conducted in patients with metastatic cancers (renal, breast or lung) reported mTOR inhibitor-related pneumonitis with a large variation in incidence [163–165].

For clinical practice, no specific recommendations for antimicrobial prophylaxis or for the diagnostic approach to individual patients with fever emerging under treatment with mTOR inhibitors can be given. In light of their overall increased risk of infectious complications, a high level of alertness is required. In patients who develop cough and/or dyspnea, drug-related interstitial lung disease should be taken into consideration.

### Ruxolitinib

Ruxolitinib is an inhibitor of Janus kinases (JAKs), which are non-receptor tyrosine kinases mediating signal transduction induced by cytokines. JAK2^V617F^ mutation results in constitutive activation of the JAK/STAT (signal transducer and activator of transcription) signaling pathway. Ruxolitinib alleviates constitutional symptoms of myelofibrosis (MF) by downregulating interleukin (IL)-1b, IL-6 and TNF-α. Ruxolitinib was approved for treatment of advanced MF and Polycythaemia Vera (PV).

Until now, three possible mechanisms of ruxolitinib immunomodulatory effects and immunosuppressive action have been identified, mainly based on dendritic, T- and natural killer (NK) cells. The first mechanism is the ruxolitinib-induced effect on DCs differentiation and function in vitro and in vivo blocking DC development [166]. In the presence of ruxolitinib, the cells morphologically and phenotypically resemble monocytes rather than DCs, and IL−12 cytokine production, which is critical for naive CD8-positive T-cell activation to acquire cytotoxic activity and to destroy infected or transformed cells, is markedly reduced. Finally, proper DC migration to secondary lymphoid organs, in order to induce T-cell responses, is also severely reduced [167].

The second mechanism involves T-cells. JAK/STAT-signaling is involved in the regulation of CD4-positive T cells, which mediate inflammatory responses and protect against a wide range of pathogens by adopting a series of distinct differentiated states, i.e., T helper cell type 1 (Th1), Th2, Th17, regulatory T cells (T_regs_), etc. Ruxolitinib treatment significantly inhibits CD4+ T-cell activation and differentiation [168, 169] reducing the number of proinflammatory Th1, Th17 and T_regs_, that have also a protective role against specific viral pathogens (e.g., HSV 2, lymphocytic choriomeningitis virus, West Nile virus), some parasites (*Plasmodium spp*., *Toxoplasma gondii*) and fungal pathogens (*Candida albicans*) [170].

The third immunosuppressive mechanism involves NK cells probably because cytokine signals mediated via the JAK/STAT pathway are determinant for NK cell activation and maturation. In ruxolitinib-treated patients, NK cell numbers are drastically reduced, in part due to defective NK cell terminal maturation [171, 172], explaining the time-dependent decrease of NK cell numbers during ruxolitinib intake. Ruxolitinib therapy is associated with the reactivation of HSV and VZV infections, similar to patients with an inherited functional NK cell deficiency [172].

Infections are among the main causes of morbidity and mortality in MF, representing the cause of death in around 10% of the cases [173, 174], mainly in advanced stages of disease.

The randomized COMFORT-I study [175] comprised 309 patients with splenomegaly and intermediate-2 or high-risk IPSS who are probably more sensitive to infections due to more advanced disease. Bacterial infections and in particular urinary tract infections (9%) and VZV (1.9%) were the main infections that occurred in patients receiving ruxolitinib during randomized treatment. At 5-years follow-up [176], the most severe infections were pneumonia and sepsis at similar rates in patients treated with ruxolitinib or placebo. Over time, VZV infections occurred at higher rate in patients treated with ruxolitinib compared to placebo, but the majority of cases were single episodes grade 1 or 2. After 36 months, no other opportunistic infections occurred. Similar results were obtained in the COMFORT-II trial [177], in which ruxolitinib was compared with the best available therapy in 219 patients. Pneumonia was the only serious infectious adverse event reported (1% in the ruxolitinib group vs. 5% in the “best available therapy” group). The other infections were of grade 1−2. In the 5-year final analysis [178], with a median duration of exposure to ruxolitinib of 2.6 years, VZV infections (11.5%), pneumonia (13%), sepsis (7.9%) and urinary tract infections (24.6%) were found; however, grade 3 or 4 urinary tract infection was reported only in 1.0% of patients, VZV in 4.3%, and no trends towards an increase in the rate of sepsis were seen over time. Two cases (1%) of tuberculosis (TB) were also documented.

Other studies confirmed the predominance of bacterial and viral infections besides sporadic opportunistic infections. The ROBUST trial [179], including 48 patients with intermediate-1 and -2 and high risk, showed only bacterial infections (urinary tract infections 16.7%, respiratory tract infections 25%) or unexplained fever (12.5%), except one case of PML. There were no reports of VZV, HBV or TB. In the JUMP expanded-access trial [180], 1144 intermediate and high-risk MF patients without access to ruxolitinib outside of a clinical study were included. All-grade infections were mainly bacterial and viral and similar to those present in the registry studies. TB was seen in three patients (0.3%) and *Legionella* pneumonia in one patient (0.1%); no HBV reactivation was reported. Among patients with resistant PV and JAK2 mutation included in the RESPONSE-1 trial [181], the rate of grade 3 or 4 infections at week 32 was 3.6% and 2.7%, respectively, similar in both ruxolitinib-treated patients and the control group treated with the best available therapy; VZV infections, all of grade 1 or 2, occurred in seven patients in the ruxolitinib group (6.4%) as compared with no patients receiving standard therapy. Similar results were obtained from the randomized study RESPONSE-2 [182] assessing 149 phlebotomy-dependent patients resistant or intolerant to hydroxyurea, 74 in the ruxolitinib group versus 75 in the “best available therapy” group. Among all patients, grade 3 infections were rare (two cases in the ruxolitinib group; influenza and bronchitis) and one case (influenza) in the control group. No pneumonia or TB reactivation was diagnosed in the ruxolitinib group. Thus, ruxolitinib was not an independent risk factor for infections in this study.

A recent retrospective analysis of 507 MF patients, diagnosed between 1980 and 2014 in five Italian hematology centers [183], described the epidemiology of infections and the impact of ruxolitinib treatment in MF. One hundred and twelve patients (22%) experienced 160 infectious events (grade 3–4, 45%), more frequent in IPSS intermediate-2 and high-risk patients and in those carrying an unfavorable karyotype. The infections were mainly bacterial (78%), viral (11%, more frequent in IPSS intermediate-2/high-risk patients) and fungal (2%); also three cases of TB infection (0.5%) were diagnosed. The frequency of infections was significantly higher among the 128 patients treated with ruxolitinib (cumulative incidence rate of 6.1% vs. 3.9 per patient-year). The type and site of infections were similar to those observed in the general population, but in ruxolitinib-treated patients, the rate of infections (44% vs. 20%, *p* < 0.001) was higher compared to ruxolitinib-untreated patients, probably also because these patients were at IPSS intermediate-2/high-risk and most (61.7%) carried a large splenomegaly, the two leading risk factors identified for infections by multivariate analysis in this study. Overall, infections were fatal in 9% of the cases. Finally, in 70 patients with MF at lower risk (intermediate-1) treated with ruxolitinib [184], after a median time of 8 months from the start of ruxolitinib, infectious complications >grade 2 were 15.9%, and were mainly bacterial (with one bone TB infection) and viral infections.

Overall, these data confirm the predominance of bacterial infections, in particular in the first months of treatment (decreasing along treatment exposition) as well as in patients who did not respond to ruxolitinib, while the VZV infection rate increased over time up to 10−11%; infections were mostly of grade 1−2. Some authors propose that prophylaxis with antiviral drugs could be considered in case of previous history of Herpes virus disease. Moreover, the immunosuppressive effects of ruxolitinib may have played a role in isolated cases of serious opportunistic infections [185–196], such as PML [77], toxoplasmosis [186], CMV [187], cryptococcosis [188–190], PcP and other fungal infections [191–193], EBV [194, 195], VZV meningoencephalitis [196] and, more frequently, reactivation of HBV and TB.

The widespread use of molecularly targeted drugs with immunosuppressive or immunomodulating action has increased the risk of HBV reactivation, which may clinically vary from an asymptomatic replication to severe hepatitis and even fatal hepatic failure. The actual incidence of HBV reactivation following ruxolitinib therapy is unknown, because most clinical trials excluded the enrollment of patients with active HBV. Until now, five case reports are described in the literature [197–200], highlighting the importance of close monitoring of liver function tests and plasma HBV-DNA level in HBV carriers receiving ruxolitinib therapy. Recently published guidelines [201] recommend HBV-screening for hematologic patients scheduled for chemotherapy and/or immunotherapy for both HBV reactivation and HBV risk factors as the first step in preventing reactivation. Screening should include HBsAg, anti-HBc and anti-HBs, and HBV-DNA if anti-HBc is positive. HBV-seropositive individuals should be started on antivirals in a timely manner. Recent guidelines [36, 202, 203] recommend the use of antiviral drugs with a higher barrier to resistance rather than lamivudine for first-line treatment. Entecavir and tenofovir are now preferred because of their lower viral resistance rates. The Centers for Disease Control and Prevention (CDC) have recommended routine postvaccination tests for anti-HBs and annual booster doses for sustained immunity among high-risk groups and immunocompromised individuals. Careful assessment of HBV infection is required before starting ruxolitinib, and monitoring of HBV markers and prophylaxis might be required for any patients that demonstrate an HBV infection during the treatment course [204].

The notification of TB cases in registry data [177, 178] and other studies [180, 183, 184] as well as case reports [205–213] have suggested a causative role of ruxolitinib in the emergence of tuberculosis. Before ruxolitinib treatment, an accurate TB history should be always taken, and the screening for latent TB must be considered if epidemiological risk factors are significant (history, endemic areas, trips in endemic areas) with Tuberculin Skin Test (TST) or (preferably) IFN-γ Release Assay, IGRA (i.e. QuantiFERON test) [204, 208]. After commencing ruxolitinib, regular follow-up of patients is advised, especially for the first 6 months, to assess for the development of opportunistic infections and TB reactivation. In the TB case reports, anti-infectious treatment was effective in most patients and, if clinically indicated, ruxolitinib was successfully resumed [207, 208] after infection eradication, resulting in MF improvement with no TB relapse.

In conclusion, ruxolitinib-treated patients should be carefully evaluated for serious infections at the onset of fever. Age and comorbidities, treatment modalities (such as glucocorticosteroids), IPSS score [214] and environmental exposure may further influence the risk of infections. Main reported infections are bacterial, in particular urinary tract infections, pneumonia, sepsis, and viral, in particular VZV infection and influenza, but ruxolitinib was also associated with a potentially increased risk of opportunistic infections. As reported in a recent meta-analysis regarding ruxolitinib- associated infections [215], severe infections may delay the eligibility of MF patients to allogeneic transplantation, so a careful evaluation of the risk of infections is recommended before ruxolitinib treatment.

HBV reactivation was occasionally seen in patients with previous history of hepatitis and/or with occult infection. Before ruxolitinib treatment, HBV screening in all patients and prophylaxis preferably with entecavir in patients HBsAg-positive and/or anti-HBc-positive is recommended. Screening for latent TB should be considered if epidemiological risk factors and medical history are significant.

In case of fever after ruxolitinib discontinuation, the possibility of a rare “ruxolitinib withdrawal syndrome”, a syndrome presenting respiratory distress, progression of splenomegaly, fever or pruritus, mimicking an infection, should be considered [216, 217].

### Venetoclax

Venetoclax is a potent and specific inhibitor of the antiapoptotic BCL-2 protein. It has been approved for the treatment of B-CLL (as third-line therapy or as second-line therapy in case of 17p deletion or TP53 mutation), where it has been shown to induce a rapid apoptosis of CLL cells, known to be BCL-2 dependent [218].

The only immunosuppressive effect associated with venetoclax is related to cytopenias. High-grade neutropenia in particular has been shown to be a common adverse effect in phase I and II studies in CLL [219, 220].

The relative role of venetoclax in this setting has been questioned, as pretreatments and marrow infiltration by CLL may have a substantial impact. Neutropenia occurs mainly during the first 3 months of treatment, and an inverse correlation has been shown between blood venetoclax concentration and risk of neutropenia and infection [221]. Improvement may therefore be related to bone marrow clearance from B-CLL. However, the causal role of venetoclax is highly probable. Venetoclax has been shown to suppress granulopoiesis in vitro and in animal models [222]. Moreover, comparative data from a phase 3 trial comparing rituximab-venetoclax to rituximab-bendamustin in relapsed or refractory CLL have shown a higher rate of grade 3 or 4 neutropenia in the venetoclax group (57.7% vs. 38.8%) [223]. Interestingly, cyclic administration of venetoclax (1 week on therapy, 3 weeks off) was not associated with neutropenia in a study on venetoclax use in systemic lupus erythematosus [224].

The real risk of infections associated with venetoclax in patients with B-CLL is unknown. In an aggregated safety analysis including one phase 1 and two phase 2 studies of venetoclax monotherapy in relapsed or refractory B-CLL, the drug has shown a manageable safety profile. Grade 3 or higher overall infection rate was 19% [225]. Reassuringly, although neutropenia was more frequent, a lower rate of grade 3 or 4 febrile neutropenia (3.6% vs. 9.6%) and grade 3 or 4 infections (17.5% vs. 21.8%) was reported with rituximab−venetoclax in comparison to rituximab−bendamustin [223].

Neutropenia has usually been managed with dose reduction or transient interruption, and G-CSF has been used with good response [220, 226]. According to the manufacturer, treatment should be withheld in case of grade 4 hematologic toxicity or in case of grade 3 or 4 neutropenia with infection or fever [227]. Infection without neutropenia has seldom led to venetoclax interruption or dose reduction [220].

Venetoclax is a substrate of CYP3A, raising concerns about the impact of CYP3A inducers or inhibitors, such as azole antifungal agents. The impact of posaconazole coadministration has been well studied, and venetoclax dose should be reduced by at least 75% [228].
